# Biogenic concrete protection driven by the formate oxidation by *Methylocystis parvus* OBBP

**DOI:** 10.3389/fmicb.2015.00786

**Published:** 2015-08-03

**Authors:** Giovanni Ganendra, Jianyun Wang, Jose A. Ramos, Hannelore Derluyn, Hubert Rahier, Veerle Cnudde, Adrian Ho, Nico Boon

**Affiliations:** ^1^Laboratory of Microbial Ecology and Technology, Department of Biochemical and Microbial Technology, Ghent UniversityGent, Belgium; ^2^SIM vzwZwijnaarde, Belgium; ^3^Magnel Laboratory of Concrete Research, Ghent UniversityGent, Belgium; ^4^Research Group Physical Chemistry and Polymer Science, Vrije Universiteit BrusselBrussels, Belgium; ^5^Department of Geology and Soil Science – PproGRess, Centre for X-ray Tomography, Ghent UniversityGhent, Belgium; ^6^Department of Microbial Ecology, Netherlands Institute of Ecology (NIOO-KNAW)Wageningen, Netherlands

**Keywords:** biomineralization, concrete protection, methane-oxidizing bacteria, formate oxidation, autoclaved aerated concrete

## Abstract

The effectiveness of Microbiologically Induced Carbonate Precipitation (MICP) from the formate oxidation by *Methylocystis parvus* OBBP as an alternative process for concrete protection was investigated. MICP was induced on Autoclaved Aerated Concrete (AAC), the model material, by immersing the material in 10^9^ *M. parvus* cells mL^−1^ containing 5 g L^−1^ of calcium formate. A 2 days immersion of the material gave the maximum weight increase of the specimens (38 ± 19 mg) and this was likely due to the deposition of calcium carbonate, biomass, and unconverted calcium formate. The solid deposition mainly occurred in the micropores of the specimen, close to the outer surface. A significantly lower water absorption was observed in the bacterially treated specimens compared to the non-treated ones (up to 2.92 ± 0.91 kg m^−2^) and this could be attributed to the solid deposition. However, the sonication test demonstrated that the bacterial treatment did not give a consolidating effect to the material. Overall, compared to the currently employed urea hydrolysis process, the formate-based MICP by *M. parvus* offers a more environmentally friendly approach for the biotechnological application to protect concrete.

## Introduction

Building materials (i.e., natural stones and concretes) are susceptible to physical, chemical, and biological weathering processes leading to the deterioration of the materials (Saiz-Jimenez, 1997). Building materials deterioration adversely affects the mechanical integrity of these materials, which in turn, decreases their lifespan (Achal et al., 2011; Labus and Bochen, 2012). Therefore, several conservation techniques have been applied to protect existing building materials (Cnudde et al., 2004; De Belie, 2010). These conservation techniques focus on the application of water repellents or consolidators with the desire to preserve cultural heritage. While water repellents are applied to decrease the rate of stone decay, consolidants are intended to strengthen decayed stone. As water repellents, inorganic/organic components have been applied previously to protect building material (Price et al., 1988; Tiano, 2004). However, these treatments pose several shortcomings such as different thermal expansion coefficient of the treated layers and the need for constant maintenance (Brajer and Kalsbeek, 1999; Murray, 2013). Thus, pore blockering products are typically applied as an alternative treatment to decelerate the impregnation rate of moisture to delay the material's weathering.

Microbiologically Induced Carbonate Precipitation (MICP) has been applied as an alternative driving process to generate pore blockers to protect concrete (see review (De Muynck et al., 2010a) and references therein for further explanation). MICP is the production of solid carbonate minerals (e.g., calcium carbonate) as a result of microbial activities. The resulting deposition of the minerals on concrete resulted in the significant decrease of capillary water uptake rate into the material (De Muynck et al., 2008a). Hence, this biogenic treatment could improve the durability of concrete (De Muynck et al., 2008b; Achal et al., 2011). These results have prompted considerable interest in further investigation of MICP-based applications as concrete pore blockers (Achal et al., 2013; Pacheco-Torgal and Labrincha, 2013).

MICP can be driven by several microbial metabolic processes, namely, oxidative deamination of amino acids, organic acid utilization, and the hydrolysis of urea (Castanier et al., 1999; Stocks-Fischer et al., 1999; Braissant et al., 2003). Among these pathways, urea hydrolysis is the most investigated process as it offers several advantages such as the high rate of carbonate production by the bacteria (Hammes and Verstraete, 2002; Hammes et al., 2003). The bacterial urea hydrolysis reaction is as follows:

(1) $$\begin{array}{l} \text{CO}{\left({\text{NH}}_{2}\right)}_{2}+2{\text{H}}_{2}\text{O}\to 2{\text{NH}}_{4}^{+}+{\text{CO}}_{3}^{2-} \end{array}$$

When external calcium is provided, then calcium carbonate can be formed:

(2) $$\begin{array}{l} {\text{Ca}}^{2+}+{\text{CO}}_{3}^{2-}↔{\text{CaCO}}_{3} \end{array}$$

Subsequently, if the system is oversaturated, then calcium carbonate can be precipitated. However, there are several drawbacks when using this process which include the emission of ammonia to the atmosphere and nitric acid production. Ammonia is in equilibrium in a solution with ammonium. Furthermore, nitric acid can be produced if ammonium is nitrified further by nitrifiers that can co-exist in building materials. Ammonia emission can contribute to the environmental pollution and nitric acid presence in the building material can accelerate the deterioration of the material (De Muynck et al., 2010a). Hence, an alternative biogenic pathway should be employed for future biotechnological applications.

**Figure 1:**
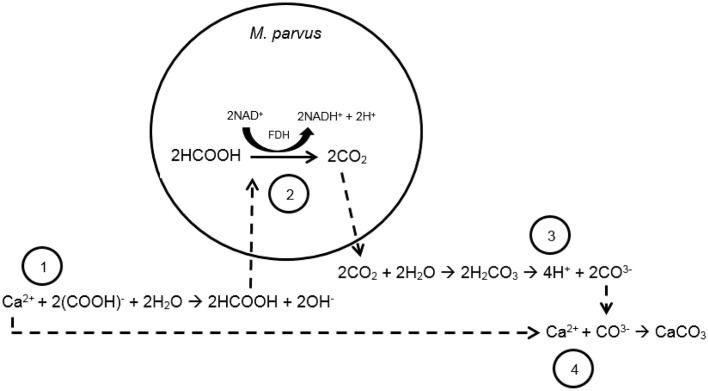
**Scheme of reaction steps of the formate-oxidation driven MICP by ***M. parvus*****. Step 1: formate dissolution into formic acid and transfer into the cytosol of the bacteria. Step 2: formate oxidation to carbon dioxide catalyzed by formate dehydrogenase (FDH). Step 3: Due to the higher local pH, higher fraction of carbonate ions in the carbonate balance is obtained. Step 4: Calcium carbonate is subsequently formed from the reaction of calcium (e.g., from calcium formate) and the carbonate ions. Calcium carbonate will likely precipitate when the system is oversaturated.

From our previous work, calcium carbonate precipitation could be induced from the formate oxidation by *Methylocystis parvus* OBBP (Ganendra et al., 2014a). *Methylocystis* spp. is Methane-Oxidizing Bacteria (MOB) belonging to the *Alphaproteobacteria* and possesses the ability to utilize methane, a greenhouse gas, as both carbon and energy sources (Whittenbury et al., 1970). The reaction steps of calcium carbonate formation driven by the bacterial formate oxidation can be seen in Figure 1. In a solution, formate is in equilibrium with formic acid. *M. parvus* oxidizes formic acid to carbon dioxide as part of their catabolic activity (Hanson and Hanson, 1996). Carbon dioxide is in equilibrium with carbonic acid, bicarbonate and carbonate ions, and the ratio of both ions is dependent on the pH of the culture. From our previous study, it was shown that the formate oxidation by *M. parvus* led to the pH increase in the culture (Ganendra et al., 2014a). The higher pH resulted in higher carbonate ions fraction from the carbonate balance. The carbonate ions can subsequently react with calcium ions, if provided externally, to form calcium carbonate. Formate based MICP could offer several advantages over the urea hydrolysis one as it does not release by-products that can pollute the environment or that are detrimental to the material.

The objective of this study is to investigate the effectiveness of formate-driven MICP by *M. parvus* OBBP as an alternative process for concrete protection. We hypothesize that the resulting calcium carbonate precipitate on concrete can protect the material by acting as pore blockers. In this research, Autoclaved Aerated Concretes (AAC) (*Ytong*, Belgium) is chosen as the model type of concrete. AAC is a lightweight porous concrete consisting of calcium silicate hydrates formed by chemical reactions between calcareous and siliceous materials (Aroni et al., 1993). AAC possesses high compressive strength and is a good thermal insulator. AAC is used mainly as wall, floor, and roof panels of residential and industrial buildings (Pytlik and Saxena, 1992). The material is selected as it exhibits a high porosity, hence, a high number of bacteria could be incorporated into the material (Samonin and Elikova, 2004). From our previous work, it was proven that higher yield of calcium carbonate precipitate over the amount of formate used could be obtained using higher amount of bacteria (Ganendra et al., 2014a).

Here, MICP evaluations on AAC using *M. parvus* were carried out. Subsequently, we characterized the AAC after MICP, and determined the effectiveness of the bacterial treatment as pore blockers.

## Materials and methods

### Microorganism and growth conditions

*Methylocystis parvus* OBBP was obtained from Colin Murrell (School of Environmental Science, University of East Anglia) on nitrate mineral salt agar plate. Prior to the experiments, the strain was transferred from the agar plate and grown in liquid nitrate mineral salt medium (Whittenbury et al., 1970) under ~20% (v/v) methane/air atmosphere in sterile serum bottles (Schott Duran, USA). The bottles were subsequently placed on a shaker (120 rpm) at 28°C. Prior to the bacterial transfer, the serum bottles were sterilized by autoclaving the bottles at 120°C for 20 min.

### Building materials

AAC blocks were cut into specimens with dimensions according to the type of the experiment as follows: (i) prisms of 2 × 2 × 4 cm (MICP on AAC and sonication tests), (ii) prisms of 3.5 × 2.5 × 1.5 cm (thin section analyses), (iii) cylinders of 1 cm in height and 0.6 cm in diameter [scanning electron microscopy (SEM) and micro-tomography analyses], and (iv) cubes with 4 cm side (water absorption and drying behavior tests). Specimens were dried at 70°C in a ventilated oven and weighed daily until constant weight was achieved [i.e., the weight difference was less than 0.1% (w/w)].

### MICP on AAC

#### MICP treatment procedure

The experiment was performed by immersing AAC samples in *M. parvus* culture containing calcium formate. The setup was prepared aseptically under laminar flow. *M. parvus* was grown in serum bottles to mid-logarithmic phase before the cells were collected by centrifugation at 11,000 × g for 20 min. The cells were subsequently washed two times with saline solution (8.5 g NaCl L^−1^) and resuspended in 5 g L^−1^ of calcium formate until a culture density of approximately 10^9^ cells mL^−1^ was reached. These conditions gave the maximum yield of calcium carbonate precipitate [g CaCO_3_ Ca(COOH)^−1^_2_, Ganendra et al., 2014a]. Bacterial cell counts were done using 50 μL portion of the resuspended culture.

AAC specimens were placed into empty 150 mL plastic vessels (Novolab, Belgium) and fixed horizontally using double-sided tape. The specimens were UV-sterilized prior to the experiment. The resuspended bacterial culture was poured into the vessels until the specimens were completely immersed. The vessels were subsequently closed and incubated statically at 28°C. The influence of the immersion period on the solid deposition on the specimens was investigated by immersing different specimens for 1, 2, 4, or 9 days. Before and after the immersion, 2 mL of liquid was sampled and filtered using a 0.22 μm pore size filter (Millipore, Belgium). The liquid samples were subsequently stored at 4°C until further analysis. After the immersion, 5 μL of liquid was taken from each vessel for bacterial cell counting before the liquid was poured out of the vessel. Specimens immersed in sterilized calcium formate and specimens immersed in calcium formate containing autoclaved bacteria served as the two controls. Experiments were done in quadruplicate.

#### MICP parameters evaluations

MICP on AAC was evaluated by assessing the following parameters: (a) specimens weight increase, (b) soluble calcium and (c) formate removal in the liquid, and (d) pH increase in the liquid.

##### Specimens weight increase

To investigate the influence of MICP on the specimens' weight, after the liquid was poured out of the vessels, the specimens were removed and dried at 70°C in a ventilated oven. They were weighed daily until the weight differences were less than 0.1% (w/w). The weight increase was calculated as the difference between the weight of the specimens before and after the treatment.

##### Liquid sample analyses

The liquid samples were used to analyze the: (i) calcium concentration, (ii) formate concentration, and (iii) pH. Samples from different treatments were diluted 10 and 1000 times for formate and calcium concentration analyses, respectively. A 1 mL diluted sample was analyzed for the formate concentration using a DX-500 BioLC liquid chromatograph that was equipped with an AS1 column and an ED50 conductivity detector (Dionex, USA). A 10 mL diluted sample was used to measure the soluble calcium concentration using an AA-6300 atomic absorption spectrophotometer (Shimadzu, Japan). Before the calcium concentration analysis, 100 and 200 μL of 65% (v/v) nitric acid (VWR, Belgium) and 1 g L^−1^ of lanthanum standard solution (Chem-Lab, Belgium), respectively, were added into each sample. The pH was measured using a C-532 pH electrode (Consort, Belgium).

##### Bacterial cell counts

Bacterial cell counts were performed to obtain the culture densities before and after the treatment. Fluorescent dye (5 μL; the composition is described in De Roy et al., 2012) and sterile physiological solution (490 μL; 0.9% (v/v) NaCl) were added to each 5 μL bacterial culture taken from the vessels and mixed. The cell counts were performed on the mixture using a BD Accuri C6 flow cytometer (BD Biosciences, Belgium) according to the live/dead staining method described by Van Nevel et al. (2013). The total number of propidium iodide and SYBR green-tagged cells per mL of the analyzed sample was reported as the culture density.

### AAC characteristic evaluations

The influence of MICP on the morphology of AAC specimen was investigated by means of: (i) SEM, (ii) thin section, and (iii) X-ray micro-tomography analyses. Based on MICP parameters evaluations (see Results and Discussion Section), 2 days specimens immersion in *M. parvus* culture was chosen as the optimum MICP method. The treatment procedure was performed as described previously and the specimen was characterized before and after the treatment.

#### SEM analyses

For SEM analyses, the specimen was placed on an aluminum stub with carbon conductive tab before analysis. SEM was performed on the specimen using a Phenom ProX desktop scanning electron microscope with 5 kV accelerating voltages (Phenom-World BV, Eindhoven, Netherlands).

#### Thin section analyses

Petrographic analyses of AAC specimen were outsourced to GEOS, an ISO 17025 accredited laboratory for concrete analyses, in Wellen, Belgium. The specimen was prepared and analyses were performed according to ASTM C 825.

#### Micro-tomography analyses

##### Sample scanning

AAC sample was scanned using the X-ray micro-tomography cone beam setup of the HECTOR scanner (Masschaele et al., 2013) at the Centre for X-ray Tomography of Ghent University (UGCT) (Masschaele et al., 2007). A total of 2401 projections were acquired from the specimen over a 360° angle with an exposure time of 1 s per projection. A thin aluminum filter (0.1 mm) was used to block low-energetic X-rays at the source to reduce beam hardening (Cnudde and Boone, 2013). In order to correct for inhomogeneities of the detector and the beam, 30 dark-field (no X-ray beam) and 40 flat-field (no sample between source and detector) images were acquired. The X-ray tube provided a voltage of 90 kV with a target power of 10 W. The source-detector and source-object distances were 1166 mm and 29.6 mm, respectively, resulting in a 5^3^ μm^3^ voxel size. The same acquisition parameters (e.g., number of projections, exposure time, filter, etc.) were used for the scans before and after the treatment. After the acquisition, the raw data were reconstructed using Octopus (Inside Matters BVBA, Belgium; Vlassenbroeck et al., 2007). The same set of parameters for ring and spot removal, tilt and skew of the detector and beam hardening were adopted for both scans.

##### Image processing

The two datasets, i.e., prior to and after the bacterial treatment, were loaded in the software DataViewer (Bruker MicroCT). The datasets were aligned manually and subsequently an automatic registration procedure was performed. This allowed to assess changes between the pre- and post-treated state of the sample by subtracting the volume of the pretreated state from the volume of the post-treated one. At locations where the likely solid precipitation occurred, a change of the gray value was expected in the X-ray images. The differential volume obtained from the digital image subtraction represented the likely solid precipitation in the sample.

### Evaluations of MICP for concrete protection

The evaluations were performed on the specimens after 2 days specimens immersion in the bacterial culture using the method described previously. Non-treated specimens and specimens immersed in calcium formate containing autoclaved bacteria served as the two controls. All tests were performed in triplicates.

#### Capillary water absorption

The aim of the test was to investigate the effectiveness of MICP on the specimens against the transport of water, the model weathering agent, into the material. After the MICP treatment, the specimens were dried and if constant specimens weight was observed, water absorption test was performed according to the EN 1925:1999 method described by De Muynck et al. (2011). Briefly, the specimens were immersed in water up to 1 mm deep in a water bath with the treated side being in contact with water and the non-treated sides coated with butyl tape. The specimens coating was done to prevent the evaporation of water through the coated sides during the test. At a specific time, the specimens were removed from the bath, wiped gently with a dry towel, and weighed. The sorptivity coefficient was calculated using the following equation:

(3) $$\begin{array}{l} \frac{Q}{A}=k\;\sqrt{t} \end{array}$$

where *Q* is the total absorbed water (cm^3^), *A* is the cross section of the side in contact with water (cm^2^), *t* is the time (s), and *k* is the sorptivity coefficient; *k* was calculated from the slope of the linear part of the curve (i.e., first five measurements) when *Q/A* is plotted over *t*^−0.5^.

#### Drying behavior

The drying behavior of AAC specimens was evaluated by assessing the water evaporation rate from the treated and untreated water saturated specimens. The test was performed on specimens at 20°C and 65% relative humidity using the open air desorption test as described by De Muynck et al. (2011). Briefly, water saturated specimens at the end of the capillary water absorption test were used for this test. All specimens were removed from the water bath to a plastic vessel prior to the test. The treated side (i.e., the one in contact with water) was turned upwards so that water evaporation could take place. To ensure that water evaporation occurred via the treated side, the non-treated sides were again coated with butyl tape. The drying behavior of the specimens was presented as the water weight loss over time.

#### Resistance to sonication

The consolidation effect of the bacterial treatment to the specimens was evaluated by sonication test according to the method described previously (Rodriguez-Navarro et al., 2003). Briefly, when the solid deposit was not adhered properly, the bacterially treated AAC specimens would exhibit similar weight loss to the non-treated specimens after sonication. Briefly, treated and untreated AAC specimens were subjected to 6 sonication cycles in a 37 kHz water bath (Elmasonic S 30/H; Elma GmbH, Germany) filled with demineralized water at 30°C. In each cycle, the specimens were immersed for 5 min in the water bath and afterwards, the specimens were dried in an oven at 70°C and the weight was measured daily until constant weight was achieved.

### Statistical analysis

Values presented are the means of replicates of different treatment. Error bars represent the standard deviations. The comparison of mean values, assuming normal distribution, was done using the One-Way ANOVA test (*P* = 0.05) to evaluate the significant differences between the values. Subsequent pairwise multiple comparisons tests (Holm–Sidak procedure) were performed to compare the differences between two mean values in the experiment (α = 0.05). Statistical analyses were carried out in SigmaPlot v12.0 (Systat Software Inc., USA).

## Results and discussion

### Proof of principle of MICP on AAC

**Figure 2:**
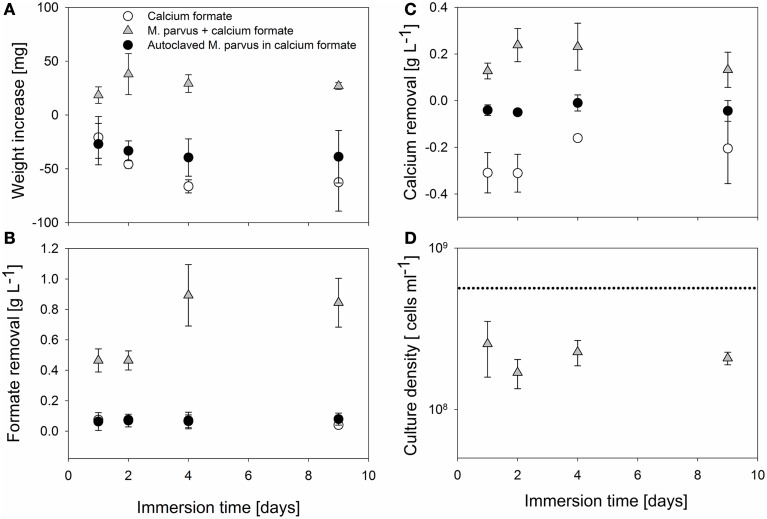
**MICP parameters evaluations on AAC immersed in ***M. parvus*** culture containing calcium formate at different immersion time**. The parameters are: **(A)** Specimens weight increase. **(B)** Formate removal in the liquid culture. **(C)** Calcium removal in the liquid culture. **(D)** Bacterial culture density.

We provide a proof of principle of the formate-based MICP on AAC and optimized the MICP method. AAC specimens were immersed in 10^9^ *M. parvus* cells mL^−1^ containing 5 g L^−1^ of calcium formate at different immersion time. Immersion in *M. parvus* culture resulted in a weight increase of the specimens (Figure 2A). Maximum specimens weight increase (38 ± 19 mg) was obtained when the specimens were immersed for 2 days. However, the difference in weight increment was not significant to specimens immersed at other time periods (*P* > 0.05). Formate and calcium removal were only observed in incubations containing live *M. parvus* cells (Figures 2B,C). Significantly higher formate removal was observed when the specimens were immersed longer than 2 days (*P* < 0.05). The highest formate removal was achieved when the specimens were immersed for 4 days (0.89 ± 0.20 g L^−1^), however, the removal difference was not significant with 9 days specimens incubations (0.84 ± 0.16 g L^−1^; *P* > 0.05). Calcium removal was observed in incubations containing *M. parvus* culture, whereas calcium release was observed in incubations in only calcium formate. Maximum calcium removal (0.24 ± 0.07 g L^−1^) was observed when the specimens were immersed for 2 days, however, the difference was not significant to the removal in other incubations (*P* > 0.05). Lower culture density was examined at the end of the immersion in all types of incubations (Figure 2D). The culture density dropped to ~ 3 × 10^8^ cells mL^−1^ from starting culture density of 10^9^ cells mL^−1^. The final culture density did not vary significantly in all incubations (*P* > 0.05).

Based on these observations, the specimens weight increase could be attributed to the calcium carbonate precipitation driven by the bacterial formate oxidation. This was inferred from the observation that the specimens weight increase was obtained only in incubations using live *M. parvus* cells where calcium and formate removals were observed. Previous studies also showed that specimens weight increase could be obtained from organic acid utilization driven MICP (e.g., acetate) on limestone (Rodriguez-Navarro et al., 2003; Zamarreño et al., 2009). Those studies used non-methylotrophic bacteria (e.g., *M. xanthus*) to drive the organic acid conversion. The specimens weight increase could also be attributed to the biomass deposition in the specimens. Based on the amount of absorbed liquid into the specimens (~10 ml per specimen) and average mass of *M. parvus* (~5 × 10^−13^ g cell^−1^, Pieja et al., 2011), bacterial cell deposition could contribute up to ~50% of the specimens weight increase. Besides producing carbonate ions and creating an alkaline environment, bacteria also play a role in the precipitation of calcium carbonate by acting as the nucleation site for the crystals (Ferris et al., 1987; Hammes and Verstraete, 2002). In our previous liquid culture study, it was observed that lower calcium carbonate purity from the formate-driven MICP by *M. parvus* was obtained suggesting the incorporation of biomass inside the crystals (Ganendra et al., 2014a). Furthermore, as formate was not fully converted by the bacteria (Figure 2B), it indicates that unconverted calcium formate could also be deposited inside the specimens. Overall, biomass, calcium formate, and calcium carbonate deposition were the likely causes for the weight increase in AAC specimens.

**Figure 3:**
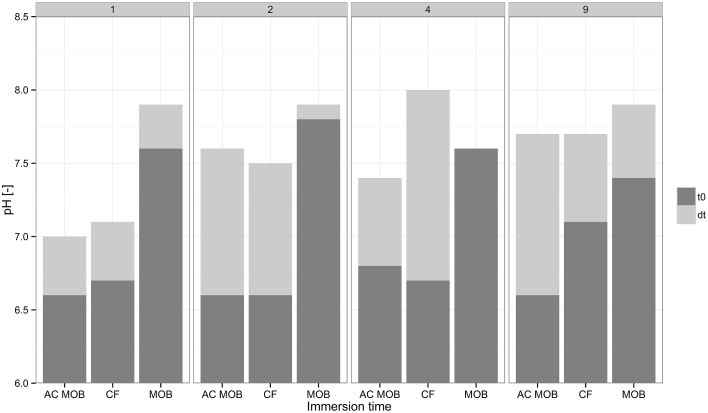
**Initial (dark gray) and final (light gray) pH in the liquid of different types of incubations containing AAC**. Different columns are the pH-values in the liquid of incubations at 1, 2, 4, and 9 days. The following abbreviations are incubations of: autoclaved *M. parvus* in calcium formate (AC MOB), calcium formate (CF), *M. parvus* in calcium formate (MOB). t_*0*_ and *dt* indicate the initial pH and the pH difference at the end of the immersion period, respectively.

AAC control specimens were dissolved during the immersion period and the effect was more pronounced the longer the specimens were immersed. Specimens immersed in calcium formate containing autoclaved *M. parvus* cells also exhibited lower weight decrease compared to specimens immersed in calcium formate only (Figure 2A). Specimens dissolution was observed from the calcium release in incubations with calcium formate only (Figure 2C) resulting in the weight decrease of the specimens (Figure 2A). Specimens dissolution was the likely explanation why the highest specimens weight increase was not achieved when the specimens were immersed for 4 days or longer. The high formate removal at these incubations didn't translate to higher specimens weight increase (Figures 2A,B). Although higher specimens weight gain due to the MICP could be achieved, the net gain might be lower due to the higher specimens dissolution. Furthermore, due to this material dissolution, higher pH was obtained at the end of the immersion period (Figure 3). AAC, a material composed of 20–40% (w/w) of calcium silicate hydrate (i.e., Tobermorite-1.1 nm; www.AAC.gr), can dissociate to calcium oxide and silicate oxide in a solution. The pH increase was the result of calcium hydroxide formation from the reaction between calcium oxide and water. Calcium carbonate precipitation could lower the pH at the end of the incubation period by shifting the carbonate balance to the production of carbonate ions and protons (Figure 1). Higher pH increase was therefore observed in the control incubations compared to the incubations with live *M. parvus* cells (Figure 3). Overall, 2 days immersion period was chosen for the subsequent experiments (i.e., AAC characterization and concrete protection evaluations) as maximum weight increase of AAC specimens could be obtained using this method.

### The influence of MICP on the morphology of AAC

**Figure 4:**
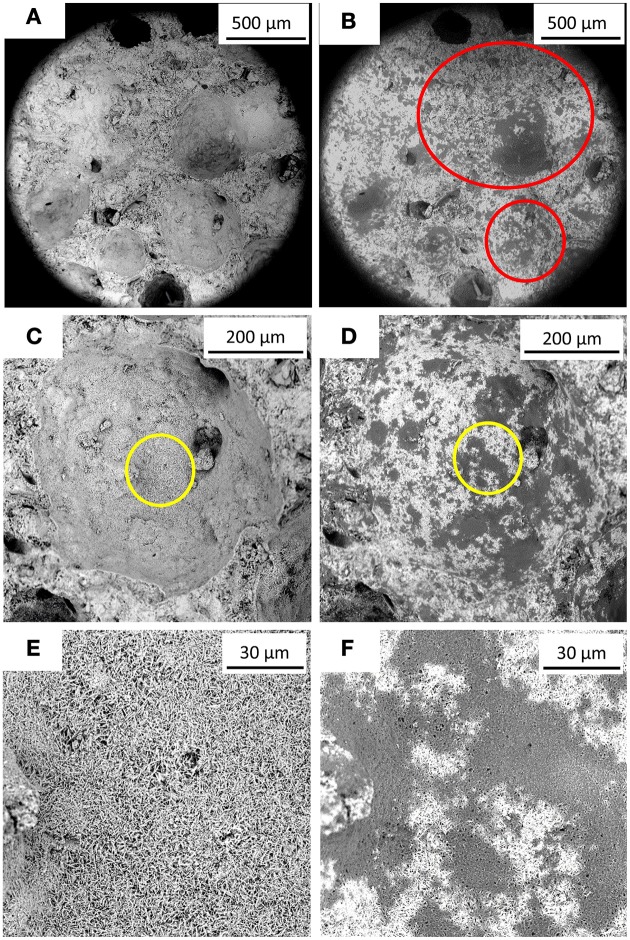
**SEM images of AAC specimen before and after immersion in ***M. parvus*** culture**. **(A,C,E)** depict different images of specimen surfaces before the treatment, whereas **(B,D,F)** depict the same surfaces after bacterially treated. Red circles in **(B)** indicate altered specimen's pore topography due to the solid precipitation. **(E,F)** depict magnified area indicated by the yellow circles in **(C)** and **(D)**, respectively.

**Figure 5:**
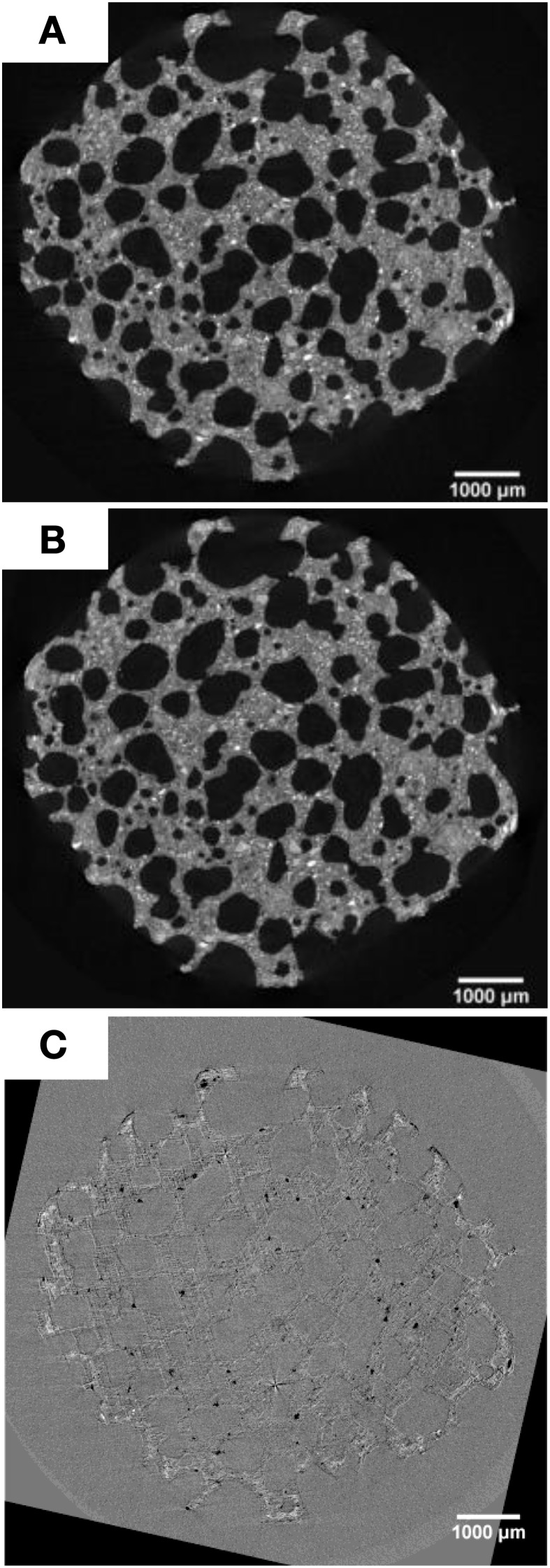
**Micro-tomographic images of AAC specimen**. Horizontal slice of the middle part of AAC specimen prior to **(A)** and after the bacterial treatment **(B)**. Image subtraction between **(A)** and **(B)** is depicted in **(C)**. White spots in **(C)** indicate the likely solid precipitation with higher density close to the outer surface.

SEM analyses showed that dark layers were exhibited on the specimen surface after the bacterial treatment (Figure 4). AAC specimen consisted of a matrix of rod-like aggregates (Figure 4E) and this dark layers filled the pores in between the aggregates (Figure 4F). Furthermore, based on the Thin Section Analysis, 5% (w/w) higher calcium carbonate fraction was obtained in the treated specimen relative to the non-treated one (data not shown). From the micro-tomographic analysis, higher solid volume was observed in the micropores of the sample, close to the outer surface (Figure 5).

The characterization of the AAC indicated that the bacterial treatment altered the micro-morphology of the specimen. The dark layers observed from the SEM analyses were most likely the solid deposition of calcium carbonate, biomass, and unconverted calcium formate. AAC consists mainly of pores with diameters of 10–100 and 0.01–0.5 μm [40% (v/v) and 55% (v/v) of the total pore volume, respectively, Ganendra et al., 2014b] and *M. parvus* could precipitate solid crystals, consisting mainly of calcium carbonate, with an average diameter of ~10 μm (Ganendra et al., 2014a). This might explain why the solid deposition only altered the microcharacteristic of the specimen.

Higher solid volume in the micropores on the specimen's outer surface observed from micro-tomographic analyses indicated that the deposition mainly occurred on this part of the material. This micro-changes cannot be observed when images prior to (Figure 5A) and after bacterially treated (Figure 5B) were visually compared. However, when looking at the difference images some changes appeared (Figure 5C). White spots can be seen in the AAC matrix close to the outer surface, which might indicate the solid precipitation. The changes are however too small, i.e., the spatial resolution is not sufficient, to perform a quantitative analysis. In addition, dark spots can also be observed. These correspond to locations where pieces of the AAC matrix have dissolved during the bacterial treatment. The microtomographic images indicate that the solid precipitate is located in the smaller pores of the AAC structure, and that the large pores remain open. No crust formation was observed, which might explain why the drying rate does not decrease after bacterial treatment (see Section The Effectiveness of MICP on AAC for Concrete Protection).

### The effectiveness of MICP on AAC for concrete protection

**Figure 6:**
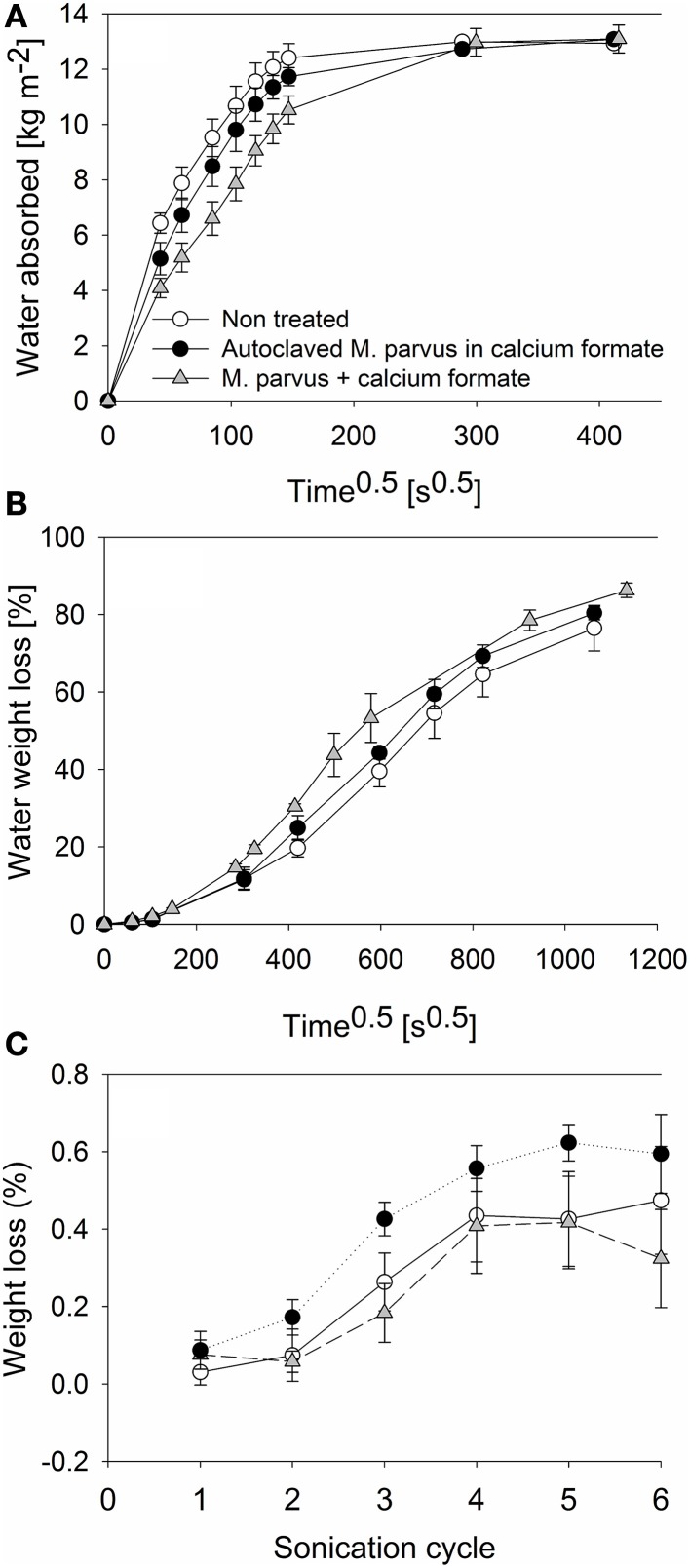
**Evaluations of the effectiveness of MICP for AAC protection**. Different evaluations are: **(A)** Capillary water absorption. **(B)** Drying behavior. **(C)** Resistance to sonication.

The experiments were performed to investigate the effectiveness of MICP on AAC as pore blockers. This was assessed by evaluating the decrease of water transport into AAC specimens after the bacterial treatment. Additionally, the drying behavior of the specimens after being saturated with water and the effect of the bacterial treatment to the cohesion of the specimens by means of sonication test were also assessed. From the capillary water absorption test, up to 2.92 ± 0.91 kg m^−2^ lower water absorption at a given measurement was observed in the bacterially treated specimens compared to the non-treated ones (Figure 6A). Lower water absorption was also observed in specimens treated with autoclaved cells. However, the difference was not significant to the non-treated ones (*P* > 0.05). Higher water sorptivity coefficient was therefore exhibited in non-treated specimens (107 ± 7 μm s^−0.5^) compared to the ones treated with autoclaved (97 ± 7 μm s^−0.5^) or live bacteria (78 ± 6 μm s^−0.5^). All specimens reached water saturated state after approximately 48 h.

Water was evaporated from all water saturated specimens (Figure 6B). The highest water evaporation rate over the test period was exhibited from specimens treated with autoclaved bacteria [4.08 ± 0.17 mg (cm^2^ h)^−1^]. Furthermore, the rate difference between the non-treated specimens [3.64 ± 0.14 mg (cm^2^ h)^−1^] and bacterially treated specimens [3.75 ± 0.18 mg (cm^2^ h)^−1^] was not significant (*P* > 0.05). All specimens showed a high initial evaporation rate (i.e., the first 4 days) but the rate decreased afterwards. The water weight loss from all specimens reached a plateau at the end of the test where ~80% of the water content in the specimens was evaporated. The highest [0.59 ± 0.10% (w/w)] and lowest [0.32 ± 0.13 % (w/w)] specimens weight losses (i.e., the difference between the specimens weight prior to the test and after 6 cycles of sonication) were exhibited by specimens treated with autoclaved and live bacteria, respectively (Figure 6C). However, the weight loss difference between non-treated specimens [weight loss = 0.47 ± 0.14% (w/w)] and specimens treated with live bacteria was not significant (*P* > 0.05).

The lower water absorption by the bacterially treated specimens could be attributed to the increased resistance caused by the solid deposition on the specimens. The capillary water absorption behavior of a material is dependent on the porosity and geometry of the material (Dick et al., 2006; De Muynck et al., 2008b). From SEM and micro-tomography analyses, it was observed that the solid deposition had blocked the specimens' micropores and this had likely caused the slowing down of the water intrusion into the specimens. Furthermore, the insignificant difference of the water evaporation rate between the non-treated and bacterially treated specimens could be attributed to this deposition in the micropores. Hence, no plugging of the macropores (i.e., pore diameter ≥ 100 μm) occurred. From the 3D micro-tomographic analyses, it was observed that the large pores of the specimen were well-connected, whereas only 1% of the resolved porosity was found to be closed pores. The high initial water weight loss could be attributed to the water evaporation from this macropores. This was the likely explanation why the water evaporation rate from the non-treated and bacterially treated specimens was similar (*P* > 0.05).

Sonication test is a standard test established by previous studies to investigate the consolidation efficacy of bacterial carbonate deposition on building materials (Rodriguez-Navarro et al., 2003; Jimenez-Lopez et al., 2007; De Muynck et al., 2010b). MICP on building materials results in the material pores filled by the biogenic calcium carbonate precipitate. This, in turns, consolidates the building material and strengthen the material from disintegration due to external forces (i.e., from the weathering processes). Using sonication test, this external forces are simulated by applying sound energy to the building materials immersed in a water bath. The adherence of the material aggregates can be known as water in the bath is agitated. From previous studies, it was demonstrated that significantly lower specimens weight loss was observed after the specimens were bacterially treated (Rodriguez-Navarro et al., 2003; Jimenez-Lopez et al., 2007). In those studies, urea hydrolysis or oxidative deamination of amino acids were the microbial processes used. After 6 cycles of sonication, it was shown in this study that lower specimens weight loss was also obtained compared to the non-treated specimens (Figure 6C). However, the consolidating effect of the solid deposition was not appreciable as the weight loss difference of the bacterially treated specimens to the non-treated ones was not significant (*P* > 0.05). This could be attributed to the low amount of solid deposition in the bacterially treated specimens (Figure 2A). Furthermore, in agreement with Jimenez-Lopez et al. (2007), higher specimens weight loss could be obtained in specimens treated with autoclaved cells due to the ease of organic matrix deposition removal (i.e., cell debris) when subjected to the test. Overall, solid deposition on AAC specimens increased the resistance of the material from the ingression of water into the specimens without consolidating the material itself.

### General considerations and outlook

This study presents an alternative MICP approach to protect concrete using *M. parvus* and formate oxidation as the microorganism and driving process, respectively. Effective MICP applications are typically based on the bacterial urea hydrolysis where, besides driving calcium carbonate precipitation, several undesirable by-products such as ammonia and nitric acid are also produced (De Muynck et al., 2010a). Ammonia release can cause human health concerns such as eye and lung irritation due to the formation of particulate matters (Winiwarter and Klimont, 2011; Hertel et al., 2012). Ammonia deposition in water bodies (e.g., lake) can also cause eutrophication (Behera et al., 2013). Furthermore, reaction between nitric acid and calcite can produce calcium nitrate, a highly soluble component, and the dissolution of this material can increase the material porosity (Piqué et al., 1992). From the environmental aspect, by employing formate oxidation, ammonia, and nitric acid are not produced. Formate oxidation-based MICP is therefore the less pollutant emitting biological process compared to the urea hydrolysis for concrete protection.

Several comparisons can be made between the results obtained in this study and other studies employing urea hydrolysis. Using lower substrate concentration (5 g L^−1^ of calcium formate), lower specimens weight gain was obtained in this study (2.4 ± 1.2 mg per cm^3^ of specimen; 2 days immersion). A maximum of ~500 mg of limestone weight increase (50–150 mg per cm^3^ of limestone) was obtained by De Muynck et al. (2010b) using 20 g L^−1^ of urea and 50 g L^−1^ of calcium chloride (De Muynck et al., 2010b). For application on concrete, De Muynck et al. (2008b) obtained a maximum of 77.1 ± 3.8 mg specimens weight increase after one time biodeposition treatment using 10 g L^−1^ urea (De Muynck et al., 2008b). As larger crystals could be obtained using a high starting substrate concentration (20–100 μm diameter; De Muynck et al., 2010b, 2011), the urea-based bacterial treatment could alter the macroporosity of the building material.

Although a low amount of solid deposition was obtained in this study, an effective material protection could still be achieved. From the capillary water absorption test, a significantly lower water intrusion rate into the material was observed when the material was bacterially treated (Figure 6A). With a higher amount of deposits in the urea hydrolysis-based studies, a significantly lower water intrusion rate into the treated building materials was also achieved compared to the non-treated ones (De Muynck et al., 2010b, 2011). In our study, up to 2.92 ± 0.91 kg m^−2^ (~30%) lower water absorption compared to non-treated samples was observed at a given measurement (Figure 6A), whereas, Dick et al. (2006) could reach ~60% lower water absorption using 10 g L^−1^ of urea (Dick et al., 2006). Furthermore, as the solid deposition influenced only the microstructure of the material, water was evaporated with ease from the material (Figure 6B). Previous research showed that when a higher amount of calcium carbonate deposit was obtained, water was evaporated at a lower rate from the bacterially treated material compared to the non-treated ones (De Muynck et al., 2011). In that study, the material surface was almost completely blocked by the biogenic calcium carbonate layer as a result of the utilization of higher substrates concentrations. Hence, the material had a high water holding capacity which might increase the probability of the material dissolution. This gives an advantage when using the formate-oxidation based MICP by *M. parvus* as an alternative biogenic treatment.

However, unlike the urea-based approach, the formate-based approach did not give consolidating effect to the treated material. This was demonstrated in the sonication test where the specimens weight loss between the treated and non-treated samples was not significant (*P* > 0.05; Figure 6C). Consolidating effect of the calcium carbonate precipitate was typically observed when a high amount of deposits was obtained. From the urea hydrolysis studies, the newly formed solids could plug the macropores (i.e., pore diameter = 100 μm) of the specimens and consolidate the building materials (De Muynck et al., 2010b, 2011). A high amount of biogenic calcium carbonate precipitate could increase the cohesion of the building material constituents and decrease the possibility of the material to detach upon the sonication test (Jimenez-Lopez et al., 2008).

Overall, the formate-based MICP using *M. parvus* can potentially be applied as an alternative process for concrete protection that is more environmentally friendly than the urea-based approach. The formate-based treatment gave an effective protection effect to the material as lower water penetration rate into the material was observed after the treatment. Furthermore, a consolidating effect to the material was not achieved as a low amount of deposit was obtained. However, this gives another advantage of the treatment as water, the model weathering agent, could be evaporated with ease from the material. This, in turns, would decrease the probability of the material dissolution.

Future studies, should look into the following suggestions. Firstly, the use of mixed MOB culture as the biological agent should be explored. The use of mixed MOB culture could lower the operational cost related to the enrichment of the bacteria where non-aseptic procedure can be applied. Secondly, future studies should look into the application of formate-based MICP on natural stones to test the effectiveness of the process to protect this type of material. Higher compatibility of the newly formed carbonate solid compared to the results obtained in this study could be achieved if the process is applied on materials composed mostly from calcium carbonate such as the natural stones. Other evaluation techniques (e.g., gas permeability test) should also be used when evaluating the formate-based treatment by *M. parvus* to further confirm the suitability of the process.

## Conclusions

MICP was induced on AAC specimens from the formate oxidation by *M. parvus*. From different immersion periods, 2 days specimens immersion resulted in the highest weight increase of the specimens (38 ± 19 mg). This specimens weight increase could be attributed to the calcium carbonate, biomass, and unconverted calcium formate deposition. Furthermore, the solid precipitation altered the material porosity by filling the micropores of the specimen. Up to 2.92 ± 0.91 kg m^−2^ lower water absorption at a given measurement was observed in the bacterially treated specimens compared to the non-treated ones. However, based on the sonication test, no consolidation effect of the specimens could be achieved after the specimens were bacterially treated. Overall, the formate-based MICP using *M. parvus* can potentially be applied as an alternative process for concrete protection that is more environmentally friendly than the urea-based approach.

## Author contributions

GG, work conception, experimental design, experimental work, experimental analysis (MICP parameters evaluation, evaluation of MICP for concrete protection), data interpretation, manuscript drafting. JW, experimental analysis (capillary water absorption, drying behavior), data acquisition/interpretation, manuscript revision. JR, experimental design and analysis (SEM), data acquisition/interpretation, manuscript revision. HD, experimental design and analysis (micro-tomography), data acquisition/interpretation, manuscript revision. HR, experimental design and analysis (SEM), data acquisition/interpretation, manuscript revision. VC, experimental design and analysis (micro-tomography), data acquisition/interpretation, manuscript revision. AH, work conception, manuscript revision, manuscript approval, work accountability. NB, work conception, manuscript revision, manuscript approval, work accountability.

### Conflict of interest statement

The authors declare that the research was conducted in the absence of any commercial or financial relationships that could be construed as a potential conflict of interest.
